# The Expert Caregiver Intervention Targeting Former Caregivers in Finland: A Co-Design and Feasibility Study Using Mixed Methods

**DOI:** 10.3390/ijerph181910133

**Published:** 2021-09-27

**Authors:** Sarah Åkerman, Fredrica Nyqvist, Laura Coll-Planas, Annika Wentjärvi

**Affiliations:** 1 Faculty of Education and Welfare Studies, Åbo Akademi University, 65100 Vaasa, Finland; fredrica.nyqvist@abo.fi; 2 Research Group on Methodology, Methods, Models and Outcomes of Health and Social Sciences (M3O), Centre for Health and Social Care Research (CESS), Faculty of Health Sciences and Welfare, University of Vic-Central University of Catalonia, 08500 Barcelona, Spain; laura.coll@uab.cat; 3 Fundació Salut i Envelliment UAB (Foundation on Health and Ageing), Universitat Autonòma de Barcelona, 08041 Barcelona, Spain; 4 Faculty of Health and Welfare, Novia University of Applied Sciences, 65200 Vaasa, Finland; annika.wentjarvi@novia.fi

**Keywords:** caregiver, health promotion, co-design, intervention, feasibility, mixed method, volunteering, sense of coherence, life satisfaction

## Abstract

Informal caregivers face risks of social isolation. Given the high prevalence of informal caregivers in Europe, a considerable proportion of the population are also former caregivers. The Finnish Expert Caregiver intervention sought to train former caregivers to become volunteers aiming to support current caregivers through mainly peer support. The aims of this mixed method non-controlled exploratory intervention study were to assess the feasibility of the Finnish Expert Caregiver intervention by co-designing and implementing the intervention, and by assessing demand and practicality with special attention to the impact of COVID-19. The findings imply that the intervention was feasible as it resulted in a co-designed training course consisting of 30 h with 25 participants enrolling and 19 of them trying volunteering activities. The participants reported high scores on well-being at all timepoints of study, however, without statistically significant differences. The analysis of the focus group interviews revealed that the Expert Caregivers experienced the intervention as meaningful and offered them a sense of belonging with the other participants. Apart from using their caregiving past as an asset, the participants also took advantage of other personal skills and resources. Risks of adverse effects were related to the participants’ expectations on their own contribution, demanding peer support recipients, poorly functioning peer support groups, and insufficient distance to one’s caregiving past. The participants stressed the need for continuing support from intervention facilitators. Future studies with larger samples should investigate whether the effects differ between subgroups of participants and explore the perspective of the peer support recipients.

## 1. Introduction

Informal caregivers, referring to family and friends who provide usually unpaid care, are the backbone of any long-term care system in Europe [1,2]. While providing informal care may entail positive aspects [3], intensive informal caregiving is associated to various adverse effects such as depressive symptoms and social isolation [4]—effects that have been frequently reported among caregivers in Europe during the COVID-19 pandemic [5]. This study focuses on informal care targeted to older adults, here defined as aged 65 and over, with care needs. Given the high prevalence of informal caregivers throughout Europe [2,6], a considerable proportion of the population are also former caregivers, that is, caregivers who have ceased caring as the care recipient has died or been admitted to a nursing home. Negative health effects such as insomnia and depressive symptoms may prevail post-caregiving [7], but most former caregivers seem to recover from such effects within one to two years [8,9]. In fact, it is not uncommon for former caregivers to take up the caregiving role again or at least report being ready to do so if needed [10,11]. This may be partly explained by the concept of generativity [12], referring to the motivation and action to contribute to the welfare of others. Generative actions may have a positive impact on the contributor as well, as volunteering is associated to several health benefits [13,14,15,16]. Indeed, a clear focus within the World Health Organization (WHO) paradigm of healthy aging [17] is volunteering, given its positive impact on the one volunteering and the one receiving the support.

Peer support differs from other types of social support, as it involves shared experiences instead of any type of befriending activity where mutual experience is not a requirement. A recent scoping review on peer support for informal caregivers of people with dementia concluded that peer support seems to have a positive impact on the support recipients [18], although evidence on best practice is limited as intervention delivery and measured outcomes vary between studies. As for the impact on the ones offering the support, one previous study [19] in England found that the volunteers reported higher personal growth and mood in comparison to the support recipients, and that the volunteers’ levels of well-being were maintained throughout volunteering, especially for those who volunteered for a longer period. Another study [20] in England identified positive effects on well-being in terms of shared experiences and a sense of connection with others within the social and organizational network as well as emotional and practical gains. However, a risk of evoking negative emotions was also noted in the results. Similarly, a recent Norwegian study [21] exploring the experiences of trained peer supporters for caregivers of people with dementia found that the peer supporters valued the activities but also stressed the need for safeguarding their privacy and setting limits. 

Finland is one of the countries with the most rapid ageing populations [22]. In Finland, typically categorized as a Nordic welfare model, municipalities as opposed to family members are responsible for providing care for adults aged 65 and over who do not manage independently in everyday life [23]. In a comparison on populations aged 16–80, Finland was found to host more caregivers but fewer intensive caregivers (who provided care 11 or more hours per week) than the European average [6]. Nonetheless, intensive informal care is provided in Finland as well, especially by caregivers who are encompassed by the public Informal Care Allowance (ICA): a care agreement with the municipality including amongst other things an economic allowance and the right to respite care [24]. Most ICA caregivers are spouses aged 65 and over who provide intensive care equivalent to institutional care [25].

Finnish municipalities, Non-Governmental Organizations (NGO), and congregations organize information, social support, and education for caregivers partly through central government funding. These activities tend to be organized by professionals. Coordinated activities for former caregivers are rather scarce in Finland, although there are some exceptions such as examples of local peer support activities organized by the nationwide association Omaisena edelleen [26] and a training program for former caregivers to become experts by experience organized by “Polli”, which is an association for caregivers in the capital region [27]. Nevertheless, there is a need for further developing activities involving relevant stakeholders to coordinate action for adults with the experience of providing informal care to support current caregivers through peer support. Such action would potentially not only benefit current caregivers but holds the potential to promote health among former caregivers as well. 

Originally inspired by the Expert Caregiver program in Catalonia, Spain [28], the Finnish Expert Caregiver program sought to create a health-promoting intervention for individuals with experience of providing informal care to older adults. A co-design approach [29] was applied in the development and implementation of the intervention, thus bringing together relevant stakeholders to design local solutions to local problems. More specifically, the Finnish Expert Caregiver program sought to co-design, implement, and evaluate an intervention by training the participants to become Expert Caregivers and take part in coordinated volunteering opportunities to support current informal caregivers through peer support activities and spokesman assignments contributing to increased awareness of informal care in society. 

The main aim of this exploratory mixed-method study was to co-design, implement, and assess the feasibility [30] of the Finnish Expert Caregiver intervention for individuals with experience of providing informal care to older adults with care needs. This main aim was divided into the following specific aims:

- To co-design and implement the Finnish Expert Caregiver intervention with special attention to the impact of the COVID-19 pandemic;
- To assess demand in terms of actual use and perceived demand;
- To assess practicality in terms of the participants’ ability to carry out volunteering activities and the effects on the participants.

## 2. Materials and Methods

### 2.1. Research Design 

This was a non-controlled exploratory intervention study with a pre-post design. Qualitative and quantitative methods were used. We conducted baseline assessment, assessment at the end of the 4-month long training course, and assessment 13 months after baseline. 

When reporting the development of the intervention in this study, we follow the recommendations in the validated Guidance for reporting intervention development studies in health research (GUIDED) [31]. We also adhere to the items recommended in the Template for intervention description and replication (TIDieR) [32] when describing the final intervention. 

### 2.2. Setting of the Intervention

The Expert Caregiver intervention was held predominantly in the mixed rural/urban area of Ostrobothnia with 177,649 inhabitants [33] living on about 7621 square kilometers of land. Ostrobothnia is one of the healthiest regions in Finland in terms of expected life expectancy [34], but the region’s dependency ratio of 68 is higher than the national average of 62.

The intervention was held in a Swedish-speaking ethnolinguistic context. Swedish is one of the two official languages in Finland. Approximately 5% of the Finnish population is Swedish-speaking, while in the region of Ostrobothnia, the Swedish speakers constitute about 51% of the population. Swedish speakers live longer than their Finnish-speaking peers [35], and Swedish-speaking older adults in Ostrobothnia have been found to possess more social resources and to be more frequently engaged in voluntary organizations than their Finnish-speaking peers [36]. Thus, the setting of the intervention involved demographic challenges but also health and cultural advantages.

### 2.3. Intervention 

The primary goals of the health-promoting intervention were to train individuals with experience of providing informal care to an older adult to become Expert Caregivers to support current informal caregivers of older adults mainly by offering peer support.

More specifically, the initial goals of the co-design phase were to create a regional reference group consisting of five persons representing NGOs, municipalities, and current or former caregivers, to create a steering group consisting of five people in leading positions, and to co-develop a training course focusing on peer support for former caregivers. We initially planned to recruit 10–15 participants in the training course and anticipated that 10 of them would try volunteering. 

### 2.4. Study Population

The only eligibility criteria for participating in the training course was having experience of providing informal care to an older person with care needs. Participants were recruited by marketing on social media, radio, newspapers, and through information events organized in three different municipalities across the Ostrobothnia region. The events and recruitment process were organized and marketed by the project partners and further advertised by reference and steer group members working with informal caregivers.

### 2.5. Measures 

To assess the demand [30] of the intervention in terms of actual use and perceived demand, we counted the number (and when applicable percentage) of participants enrolling in the training course, completing the training course, volunteering one time, volunteering multiple times, as well as the number of organizations, congregations, or municipalities involved in the intervention (apart from the intervention providers) and finally the caregivers reached through peer support activities.

To assess practicality [30], we focused on two key concepts: ability of participants to carry out activities and effects on participants. We explored practicality from the perspective of the Expert Caregivers. We used data gathered from qualitative semi-structured focus group interviews with 13 participants collected 13 months after baseline. The participants were divided into four natural groups of three or four persons and were asked to discuss the process from caregiver to becoming an Expert Caregiver, the experiences of and perceived positive and negative effects on their well-being by volunteering as well as their experience of volunteering during the COVID-19 pandemic. A descriptive analysis (not presented here) showed that proportionally, participants of male gender, living in singe households, and holding an upper secondary education degree were slightly more represented in the focus group interviews than in the total sample. The focus group interviews were held by four project staff members: three researchers and one educational planner. The qualitative data are reported according to the standards for reporting qualitative research [37].

To further explore the effects on the participants, we used repeated questionnaires collected at baseline, at the end of the 4-month-long training course, and 13 months after baseline. The perceived effects on the participants’ well-being were explored by assessing the participants’ sense of coherence (SOC) [38] and Diener’s Satisfaction With Life Scale Index (SWLS) [39]. The scales showed a good internal consistency at all timepoints of the study with scores from Cronbach’s alpha tests ranging from 0.74 to 0.89. 

The SOC results were assessed both as a whole and divided into the three dimensions of comprehensibility, manageability, and meaningfulness. Comprehensibility is the cognitive dimension and refers to the extent to which an individual perceives his or her circumstances as understandable [38]. Manageability is the behavioural dimension and refers to the experience of possessing the resources needed to cope with current circumstances, leading on to the motivational dimension, meaningfulness, that refers to whether the individual finds motivation and meaning in facing his or her challenges. The 13-item SOC-index was to be answered on a 1–7 Likert scale. The whole SOC-index score ranges between 13 and 91 points. The subscale for meaningfulness has a range of 4–28 points, that for comprehensibility has a range of 5–35 points, and that for manageability has a range of 4–28 points. Higher scores indicate higher SOC.

The SLWS assesses life satisfaction and is a 5-item questionnaire to be answered on a 1–7 Likert scale [39]. Higher scores indicate higher satisfaction, so that 30–35 indicates very high score, 25–29 indicates a high score, 20–24 is average, 15–19 is slightly below average, 10–14 is dissatisfied, and 5–9 is extremely dissatisfied. The quantitative data were collected on site at all three timepoints of study and sent out by post to those not present.

### 2.6. Analysis

The qualitative focus group interviews were recorded and transcribed verbatim by one of the researchers (Sarah Åkerman). The same researcher (Sarah Åkerman) divided each transcript into inductively chosen categories. The analysis of the focus group interviews was interpretative–phenomenological [40], meaning that the researchers are active in the dynamic research process, aiming to help the participants make sense of their personal and social world. Interpretative phenomenological analysis usually involves small sample sizes from heterogeneous populations, as the aim is to investigate the meaning of a certain group’s experiences of a specific phenomenon or event. 

Initially, the data were scrutinized into multiple categories by Sarah Åkerman Step by step, the categories were reread and reorganized into broader themes sharing common patterns. For the analysis, the software program of NVivo 12 (QSR International, Doncaster, Australia) was used. To ensure trustworthiness of the findings [41], two other researchers (Fredrica Nyqvist and Annika Wentjärvi) then independently read through the transcribed interviews and compared the data with the coding schemes. Finally, the three researchers discussed the coding scheme until agreement was reached. 

For the analysis of the quantitative data, the distribution of all the participants’ variables was calculated. Statistical analyses were conducted to assess the mean, range, standard deviation, and median for the participants’ SOC and SLWS scores at all three timepoints. 

To report differences between the three timepoints of study (baseline, 4 months after baseline, and 13 months after baseline), the participants’ average scores on SOC and SLWS were compared. Variables with a normal distribution (Kolmogorov–Smirnov test with *p* ≤ 0.05) were analyzed with a one-way repeated ANOVA to compare the differences in average scores between the timepoints. Variables without a normal distribution were analyzed with the Friedman test. All statistical analyses were performed in the statistical program IBM SPSS Statistics 27 (IBM Corp, Armonk, NY, USA).

### 2.7. Ethical Considerations

The study follows the Guidelines of the Finnish Advisory Board on Research Integrity TENK [42]. Ethical committee approval was not deemed by members of the experienced researcher team to be needed for this non-medical study where we evaluated an intervention focusing on education and knowledge transfer. The study subjects were all adults and participated in the research elements voluntarily after being informed about the aim of the research, data management, and use. The intervention required active participation from the study subjects, and therefore, they were deemed capable of making informed decisions about taking part in the intervention and research activities. Furthermore, the participants enrolled voluntarily in the intervention, comprising of a training course and coordinated volunteering opportunities, and the risks of unforeseen mental or physical harmful consequences were deemed non-existent. Written informed consent was gathered from those participating in the focus group interviews. 

## 3. Results

### 3.1. Co-Design and Implementation

The Finnish Expert Caregiver Intervention in Ostrobothnia (the Finnish context is presented in the Introduction, while the Ostrobothnia region is described in Materials and Methods) was organized by two educational units (Åbo Akademi University and Novia University of Applied Sciences) and Folkhälsan, the largest NGO in the field of social and health services in Swedish-speaking Finland. The staff members from Åbo Akademi University consisted of three people: one associate professor (Fredrica Nyqvist) in Social Policy working as the project leader, evaluation planner, and researcher in the intervention, one Social Policy doctoral student (Sarah Åkerman) working as a project coordinator, researcher, and facilitator, and one educational planner with the main responsibility of the development of the training content and materials. The staff members from Novia University of Applied Sciences consisted of three people: one research leader (Annika Wentjärvi) who worked mainly as an evaluation planner and researcher, one research and development assistant who worked with administration, and finally one educational planner whose main responsibility was the training course content and materials. At NGO Folkhälsan, one staff member was involved in the intervention. She, as an expert on informal caregiving and aging at Folkhälsan, worked as a facilitator and education planner in the intervention. All staff members were involved in planning the content of the training course and the materials. 

The intervention was informed by previous research on the needs of caregivers, the potential resources of former caregivers, and the potential benefits from volunteering for both the support providers and receivers (discussed in the Introduction). Furthermore, the intervention was guided by a salutogenetic theoretical framework [38,43], as it sought to promote health by creating opportunities for the participants to bond with others in a similar situation, to reflect on the individual process from caregiver to volunteer, to reflect on personal skills, and be equipped with the appropriate tools and adequate support to be able to volunteer by offering mainly peer support. Thus, the intervention was also in line with the WHO paradigm of Healthy Aging including, amongst other things, the principles of continuing to learn, to maintain and build relationships, and to contribute [17]. 

**Figure 1:**
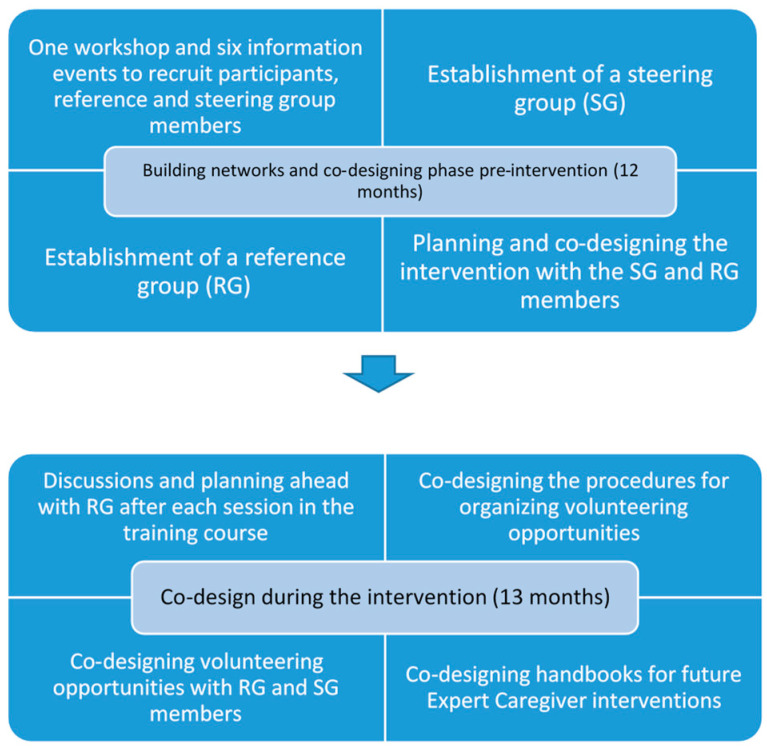
The co-design process.

A co-designing approach means the inclusion of users and producers of services at different stages throughout an intervention development to not only develop but infrastructure the new product [29]. A reference group and steering group were successfully established. Apart from staff members of the intervention, the reference group consisted of six former/current informal caregivers of older adults with care needs (of whom three participated in the intervention also as Expert Caregivers), two public social care workers from two different municipalities, one deacon, and one NGO worker. The steering members represented two NGOs and three municipalities. The co-design procedure before and during the intervention is further presented in Figure 1. Involving both Expert Caregivers and professional stakeholders in feedback discussions throughout the intervention enabled adjusting the intervention to match the local needs. 

The co-designing phase resulted in a group-based training course consisting of 30 h and 11 months of coordinated volunteering opportunities for the participants. The content of the intervention is presented in Table 1. 

In total, 25 participants answered questionnaires at baseline assessment with questions on health and background information. Of the 25 participants, 24 answered the questionnaires in the second assessment 4 months after baseline, and 23 participants filled in the questionnaires in the assessment 13 months after baseline. One participant withdrew from the program because of personal health problems, and one participant was unavailable for contact.

Out of the 23 final participants, 13 participated in the focus group interviews held on-site 13 months after baseline. The relatively low number of participants is explained by barriers to meeting on-site in September 2020 due to COVID-19.

One participant withdrew from the program after the first session of the training course because of personal health problems. One participant was unavailable for contact right after the third training course session.

As further presented in Section 3.2, 19 participants tried volunteering after the training course. The four participants who completed the training course but chose to not try volunteering were all female. The main reasons for not trying volunteering were providing informal care (*n* = 1), personal health concerns (*n* = 2), and family concerns (*n* = 1). 

Five months into the intervention, the outbreak of COVID-19 reached Finland in March 2020. National recommendations were introduced advising Finns aged 70 years and older not to meet anyone outside of the household face to face. As these recommendations applied to almost all Expert Caregivers and their peer support recipients, all physical meetings were reorganized into telephone and virtual meetings. This required extra resources from the NGO involved in the intervention as both Expert Caregivers and peer support recipients needed technological guidance. For the intervention to be adequately tested and implemented in the region despite COVID-19, the intervention was extended from the planned 9 months to 13 months.

### 3.2. Demand

Twenty-five participants enrolled in the training course, and 23 (92%) participants completed it. The characteristics of the final 23 participants are available in Table 2. 

Out of the final 23 participants, 19 participants (82%) volunteered during the intervention, and 17 (74%) volunteered at multiple occasions. All types of volunteering are included in these numbers: individual or group-based peer support activities or spokesman assignments. 

In total, 15 organizations, municipalities, eldercare units, or congregations were involved with either marketing or co-organizing the volunteering activities with the intervention providers.

The Expert Caregivers reached 60 informal caregivers through group-based peer activities and nine informal caregivers through individual peer activities. Nine Expert Caregivers performed spokesman assignments in newspapers, radio interviews, and activities organized for informal caregivers and/or professional stakeholders.

### 3.3. Practicality: Ability to Carry Out Intervention Activities

Below, we present the analysis concerning the ability of participants to carry out intervention activities based on the focus group interviews. A summary of the results from the focus group interviews is available in Table 3.

#### 3.3.1. Individual Barriers and Facilitators to Becoming an Expert Caregiver

##### Enough Distance to One’s Own Caregiving Experience

*“It (the care recipient passing away) was about 2–3 years ago for me so I think I have the distance that is needed. As a recent widower I would not have been able to take on this assignment.” *

*Male participant*

*“It’s the same for me, it was about 2 years ago that, that I experienced… I would not have been ready before that. Then I was so tired and… I think I needed to work through the grief and move on and find myself, who am I in this? Because one has lived, my husband was ill for 10 years, I lived through him for 10 years. /…/ When you are by yourself and it all runs off you, what do you do? Building, getting a new structure on everyday life… It takes time.” *

*Female participant*

Being able to volunteer as an Expert Caregiver was affected by the participants’ distance to their own caregiving experience. While distance for some participants meant that enough time had passed since their caregiving situation ended, distance was also about the characteristics of the past caregiving situation. In general, the participants seemed to agree that providing care for a spouse with cognitive disorders was the most troublesome care context possible, which demanded more time to cope after the caregiving situation had ended. However, there were individual differences. One participant was a spousal dementia caregiver at the start of the intervention and still had the capacity to become an Expert Caregiver. 

One participant experienced that the way the caregiving situation ended had helped her cope and be at peace with the past. For her, that meant being together at home until the very end when the care recipient, her husband, passed away. Not having him admitted to a nursing home made her feel content with her caregiving experience. 

##### Current Life Circumstances

Personal health issues, family tragedies, being busy visiting one’s spouse in a nursing home, or providing informal care to someone new were reported as personal obstacles for not volunteering actively.

*“Well I had thirteen years of experience of providing care to my husband, but then I had to have surgery… I informed them (intervention facilitators) that I maybe could volunteer in the future but not at that time.” *

*Female participant*

*“Somehow I ended up in a situation where I was an informal caregiver again, unpaid, and she… It was quite hard because she got very ill for a while and was in a hospital ward here in the X (municipality) and then I was in contact with the care staff daily, so I just went into that role completely. /…/ so I was not an Expert Caregiver, I was an informal caregiver.” *

*Female participant*

#### 3.3.2. Competency as an Expert Caregiver

##### Using New and Old Skills 

Several participants deemed that their potential peer support recipients would appreciate that they had been trained for the assignment. The participants also highlighted the promise of silence they had signed during the training course. They honoured this promise and knew it was important for their peer support recipients. 

The participants described using their past as an asset not only in terms of their caregiving experience but also because they got to take advantage of other skills. Several participants described using personal skills they had learned from their past working life, such as keeping emotional distance to clients or being used to talking in front of people. Participants also mentioned taking advantage of their personality traits such as being helpful and easily speaking about difficult topics or sociability.

*“I haven’t had any issues talking to people in the past either… So I’m not nervous when we get together.” *

*Male participant*

In the eagerness to help caregivers, some participants described potential difficulties with only listening to the caregiver instead of rather telling him or her what to do.

*“The issue is that one wants to say: ‘in my case, it was like this’ but you shouldn’t do that (laughs). So it’s a little bit difficult to stay put in one’s place.” *

*Male participant*

##### Relationship between Expert Caregiver and Peer Support Receiver

Personal chemistry was described as important in the relationship with the peer support receiver or group of peer support receivers. Well-functioning chemistry resulted in a more positive and boosting experience for the Expert Caregiver, while situations of poor chemistry made the Expert Caregivers self-doubt to some degree. This is illustrated in the quote below.

*“It wasn’t quite right /…/ I don’t know the reason why, if it was me or… /…/ If it’s a group in a small village, it can be difficult for the group participants to talk freely when there are others from the village around. I thought of that, that it was something… Something there that I couldn’t see. In the air.” *

*Female participant*

The training course had limited focus on how to create well-functioning personal chemistry or manage poorly functioning relationships with peer support recipients. Instead, in case of malfunctioning personal chemistry, the participants were advised to reach out to intervention organizers to end such relationships. 

The role of personal chemistry may be even more important in individual peer support relationships. One participant described her initial hesitant feelings toward becoming a peer supporter to someone known from the past but then concluded that this factor might have contributed to a relaxed peer support relationship.

*“When I became a peer supporter to this person I’m supporting, I was a bit hesitant at first because it’s an old acquaintance of mine. /…/ But it works well. And now I don’t know if it’s because I already knew her, that we can have a natural relationship.” *

*Female participant*

One participant was used to providing informal peer support to friends but experienced that for her to be able to fully step into the Expert Caregiver role, she would need a stranger as a peer support recipient.

*“I would like to have a stranger (as a peer support recipient) so that one could… Because trying to be an informal Expert Caregiver to old acquaintances is on another level.” *

*Female participant*

##### The Role of the Intervention Organizers for Feeling Safe

A few participants expressed feeling safe volunteering as an Expert Caregiver partly because of the available support from intervention professionals. One participant described how, as they are older themselves, potential peer support recipients already exist in their personal network. Having completed the course and the opportunity to dial an intervention organizer to ask for support increased the sense of safety and was experienced as a good thing also for the peer support recipient’s sake.

*“I dare to take on that role and they (the caregivers) also feel safe, those that I know. Because we get these peer support recipients automatically, we do not need to have them assigned to us, they are automatically around us. But I feel safer having you in the background, someone to talk to, I can dial X (intervention organizer) for example and discuss if there is something I’m thinking about. For the peer support recipient’s sake as well.” *

*Female participant*

While none of the Expert Caregivers had experienced such a situation yet, a few participants expressed concern about potential demanding individual peer support situations where it would be difficult to set boundaries. While some participants had faith in the intervention organizers in case of a difficult situation, one participant was not sure it would help. This is illustrated below in the conversation between two participants:

*“As long as you don’t get a person who ‘takes the whole hand’. Then you have to be quite good at setting boundaries, but it can be difficult.” *

*Female participant*

*“It can be difficult, but we have X’s (intervention organizer) support there.” *

*Male participant*

*“But it’s not certain that it helps.” *

*Female participant*

#### 3.3.3. Volunteering during COVID-19

Volunteering during COVID-19 meant peer support through virtual contact either via face video technology or telephone. One participant thought that it would have been easier to convert to virtual support if she had known the peer support recipients for a longer time and had established solid relationships. One participant was more positive about the experience, but he had been trained to offer virtual peer support already before the pandemic.

*“In the beginning it was difficult with the technique, but gradually it worked out and one became used to it. /…/ So it’s sort of fun, and enables connection with others.” *

*Male participant*

Two of the participants offering individual peer support via telephone experienced that it had worked well but was not the same as offering peer support face to face.

*“It’s been, what should I say, alright but… You haven’t really been able to have the contact you meant to have, but we have been keeping in touch via telephone. It has worked well.” *

*Female participant*

*“Well the telephone works of course, but it’s not the same (as meeting face to face). *

*Male participant*

Being an Expert Caregiver during the pandemic also enabled the participants the opportunity to stay connected to other people despite the social restrictions applying to most of them. One participant described how the virtual meetings were his only social contact during the pandemic. Another participant started providing informal care again during the pandemic, and while it was not an Expert Caregiver assignment, she explained the need to be useful and socially active during the pandemic:

*“I lived in isolation but had daily social contact. For me it was a godsend that she (informal care recipient) came into my life and I got this role (as an informal caregiver).” *

*Female participant*

### 3.4. Practicality: Perceived Effects on Well-Being 

#### 3.4.1. Perceived Effects on the Participants’ Well-Being and Related Mediators

##### Expectations Influencing the Experience

An Expert Caregiver’s main task in peer support situations was to listen. Still, the levels of expectations the Expert Caregivers had put on themselves varied. Those who had moderate expectations seemed to experience greater satisfaction. One participant described the issues with having too high demands on oneself in group-based peer support situations.

*“If one starts to demand too much of oneself and think that now I don’t live up to (the caregivers’ expectations), it should be better, I should… If you put too high demands on yourself, then it becomes a torment. That I’m not enough, they must have been disappointed with the meeting, ‘what was that, just sitting and drinking coffee and talking…’. You should have those demands on yourself on a reasonable level.” *

*Female participant*

Similarly, individual peer support may also bring certain expectations that may not be fulfilled. This is illustrated in the quote below:

*“I have very little experience because I only have one adept and we have met mostly on the telephone, also physically a few times, but, but not… It has not been any counselling and that sort. /…/ So there has not been any overwhelming experiences.” Male participant*

##### The Needs of Caregivers as a Stressor

In a similar manner as to how the distance to one’s own caregiving history affected the ability to take on Expert Caregiver assignments, this distance also affected the well-being outcomes of volunteering. Not all participants experienced being at peace with the past yet. One participant described how being in contact with a current caregiver in her personal life awoke negative feelings.

*“I met someone who was in the same situation that I was in, who had just left her husband (having him admitted to a nursing home), and I felt so bad the entire evening when she told me, because I went back to how it had been for me. I thought about her all night after she told me.” *

*Female participant*

Knowing the needs of caregivers contributed to some participants feeling stressed about all the things they wanted to improve, such as issues with caregivers who suffer but choose not to reach out for support.

*“The only negative thing is that more people should take advantage of peer support, that you would really get it out there to those needing it, because the needs are actually extensive.” *

*Female participant*

*“There are a lot of people in X (municipality) who should get in touch with us, but they don’t.” *

*Male participant*

##### Volunteering as a Therapeutic, Meaningful, and Joyful Activity

One participant thought it was therapeutic to prepare himself for group-based peer support occasions, as it not only occupied his time but made him reminisce his own caregiving experience.

*“I feel that these Expert Caregiver conversations have rewarded me in that I have had to prepare myself, I haven’t dared to go unprepared to those conversations, but I have had to look through my life a bit in advance and try to formulate the questions that need to be ventilated. That has given me something to do… and been like therapy for myself as well.” *

*Male participant*

The same participant described how offering peer support to current caregivers upset him to some degree, but that the rewarding effects still outweighed the negative.

*“It does open old wounds somewhat, but on the other hand, I think that the positive dimension outweighs it. After the discussions that I have been part of, I have been left with a good taste.” *

*Male participant*

Another participant emphasized the rewarding effects of helping others.

*“It’s also rewarding. It gives to someone who has gone through it. It heals you.” *

*Female participant*

Knowing the needs of caregivers and the, for some participants, almost traumatic experiences of informal caregiving, contributed to several participants experiencing a sense of purpose in volunteering as an Expert Caregiver.

*“I have many times thought that what if this existed when we started off as informal caregivers and one would have received this.” *

*Female participant*

One participant concluded that helping others always has a selfish side to it, as it feels nice to be needed. This was also illustrated in another participant’s quote on the experiences of a group-based peer support event:

*“And at times, I felt very important. And that was nice.” *

*Male participant*

Feeling needed also related to the participants’ past as an informal caregiver where their everyday lives revolved around helping a loved one. Now when they were no longer caregivers, and in many of the cases no longer spouses due to the death of the care recipient, one of the participants described the positive experience of being needed once again as an Expert Caregiver:

*“We have felt needed when they lived. You had to give 100% and more. You felt needed and now when they are gone you also want to be needed, so now I feel like we have a sense of purpose.” *

*Female participant*

While it was common for the participants to describe the experiences of volunteering as rewarding in terms of purposeful, a few of the participants described volunteering more as a joyful activity. This is illustrated in the quote below:

*“I like these kinds of things, so I don’t mind it. It’s not work, it’s pleasure. If you can say that, perhaps you should not say that. But I really don’t mind it.” *

*Male participant*

##### A Sense of Belonging with the Other Participants

The participants seemed to experience a sense of belonging with the other participants, regardless of whether they had been active as volunteers or not. Most of the Expert Caregivers were bereaved spousal caregivers and several participants described how the intervention provided them a longed-for appropriate social context as a widow and former caregiver. This is illustrated in the quote below:

*“And the thing is that when you become alone, there is so much where you feel like you don’t fit in, but in this context, we fit in.” *

*Female participant*

### 3.5. Sense of Coherence and Satisfaction with Life Scale

Table 4 details the average scores and changes in the SOC and SLWS scales across the three timepoints. 

For all of the SOC dimensions, an increase in the score reflects an increase in SOC. Although the scores fluctuated somewhat, no statistically significant differences between the timepoints were found in the SOC total score and its subdimensions due to small sample sizes. As all the variables in the SOC subdimension of comprehensibility were normally distributed, a one-way repeated measures ANOVA was conducted to compare the scores between the three timepoints. There were no significant effects for time on comprehensibility, as the results from the ANOVA analysis showed Wilk’s Lambda = 0.91, F = (2, 21) = 1.02, *p* < 0.38, multivariate partial eta squared = 0.09. For the rest of the SOC variables, which were non-normally distributed, the Friedman Test was used to compare the scores between the three timepoints. The result for the total SOC index showed X2 = (2, *n* = 23) = 0.64, *p* < 0.73. The result for manageability was X2 = (2, *n* = 23) = 2.85, *p* < 0.24. The result for meaningfulness was X2 = (2, *n* = 23) = 0.50, *p* < 0.78. 

A high level of life satisfaction measured with SLWS [39] was observed at all timepoints, although the median scores fluctuated somewhat. No statistically significant difference between the timepoints was found in the Friedman Test: X2 = (2, *n* = 23) = 0.64, *p* < 0.73.

## 4. Discussion

### 4.1. Interpretation of Findings

The Expert Caregiver intervention in Ostrobothnia, Finland sought to co-design a training course for 10–15 individuals with experience of providing informal care to an older adult to become “Expert Caregivers” so that 10 of them would participate in coordinated volunteering opportunities for current caregivers with an emphasis on peer support. 

The co-designing and building networks phase of the intervention resulted in the establishment of a reference group and steering group who supported the design and implementation on regular meetings throughout the process. A 30-h training course targeting individuals with experience of providing informal care was co-designed. The results showed that there was a demand of the intervention as 25 participants enrolled in the training course, despite a preliminary aim of only 10–15 participants. Furthermore, 19 participants volunteered during the intervention and 17 participants volunteered several times. Volunteering opportunities included both individual and group-based peer support meetings as well as spokesman assignments in newspapers, radio interviews, etc. Through peer support assignments, the Expert Caregivers reached 69 individual current caregivers with 15 organizations involved. The success of the intervention is to be explained by the participatory approach [29] used where the intervention departs from local needs and resources.

The intervention had to be adapted to the COVID-19 pandemic, leading to all peer support relationships being redirected to virtual or telephone contact. This required extra resources as many participants and their peer support recipients were aged 70 and over and varied in their digital skills. The Expert Caregivers experienced that offering virtual peer support worked well but estimated that this kind of support was less effective than if the support had been given face to face. However, technological solutions are increasingly emphasized for informal caregivers in Finnish national steering documents [25] as caregivers may be bound to their homes due to caregiving responsibilities. Therefore, preparing former caregivers to offer virtual peer support may be useful also in future peer support interventions. A review on the effects of virtual and face to face peer support to caregivers of people with dementia suggested that peer support may have a positive impact on caregiver well-being regardless of delivery mode [18]. 

The qualitative data gathered in the focus group interviews implied that former caregivers’ well-being may be enhanced by the Expert Caregiver intervention as it offered the participants, mostly older bereaved spouses, a social context and a sense of belonging with the other participants. The training course and the available support from the intervention organizers contributed to most participants feeling confident and safe as volunteers. When volunteering, the participants got a chance to not only use their caregiving past as a resource but also to take advantage of other personal skills and resources. Offering peer support to current caregivers offered the participants a meaningful, therapeutic, and even joyful activity. However, risks of negative effects were noted among the participants in terms of not having enough distance from one’s own caregiving experience, in situations of malfunctioning peer support groups, too high expectations on one’s contribution, or too high demands of the peer support recipient. Our results, in terms of the mainly positive impact but also risks of adverse effects, are in line with previous findings exploring the experiences of former caregivers volunteering to support current caregivers [20,21]. Indeed, in a Norwegian study, the peer supporters appreciated using their past as a resource but also experienced the need for safeguarding their free time and setting limits, as providing peer support was experienced not only as rewarding but also exhausting [21]. Similar results in terms of mostly positive effects such as emotional gains and new social contacts were reported from a peer support program in England, but the participants stressed the need for the right timing of transitioning into the peer supporter role to avoid awakening negative emotions [20]. 

The participants reported high levels of SOC and SLWS throughout the intervention, although no statistically significant differences were found across the three timepoints of study. A future study with a larger group of participants should continue to assess the effects on well-being outcomes and analyse whether this type of intervention is more suitable for certain subgroups of individuals with experience of informal caregiving. Previous research on former caregivers in general suggests that the post-caregiving experiences and outcomes may depend on, amongst other things, the characteristics of the past caregiving situation such as pre-loss levels of social support [8,9], pre-loss levels of depressive symptoms [7,9], and whether the care recipient died in the preferred setting [44]. 

It is noteworthy that most of the participants in the Expert Caregiver intervention were of female gender and holding an upper secondary education. While the evidence is not completely consistent, caregiver well-being and care characteristics seem to depend on sociodemographic features in terms of high caregiving intensity and adverse effects being more associated to female gender, lower education levels, and lower income [4,6,45,46]. Most of the participants in the Expert Caregiver intervention had provided care to someone who did not have cognitive disorders. Providing care to a spouse with cognitive disorders is generally considered the most burdensome care context [4], possibly requiring a longer time to recover from potential adverse effects. The share of male participants was small given the less gendered caregiving role among older adults [47] especially among older Finns encompassed by the public Informal Care Allowance [25]. However, previous examples of volunteering programs for former caregivers in other countries have also been more represented by female participants [19,20,21]. 

The participants in the intervention reported high scores on well-being throughout the intervention. This is in line with the results of a study in England assessing, amongst other things, the impact of a volunteering program on the peer supporters of current caregivers [19]. The fact that the participants reported high scores on well-being is also in line with previous studies on post-caregiving health in general suggesting that for most former caregivers, adverse effects associated to intensive caregiving diminish with time [8,9,45]. Still, studies have also found that for some post-caregivers, negative health effects [7,8,9,44,45] such as insomnia, depressive symptoms, and feelings of guilt may prevail, even for as long as up to 10 years [7]. The specific needs of such vulnerable former caregivers should be further studied and met in interventions where they are the end-users. Nonetheless, the Finnish Expert Caregiver intervention showed promising results in terms of training former caregivers with sufficient distance to one’s caregiving past to become volunteers to support current caregivers.

### 4.2. Strengths and Limitations

Limitations include a small sample size as well as the absence of results from a control group. Collecting questionnaires on-site could affect the validity of the quantitative data, as participants may have been influenced by the presence of other people when filling in information about their well-being. Another limitation is the exclusion of data collection from the peer support recipients about their experiences and perceived effects of the intervention. This should be further investigated in a future study. 

The implementation of the intervention was affected by the COVID-19 pandemic and causes difficulties in interpreting the results of the intervention in terms of demand and perceived effects in comparison to if the intervention had been organized in normal circumstances with an emphasis on face to face rather than technological peer support situations. On the other hand, the idea of technological solutions for supporting informal caregivers being bound to their homes because of remoteness or caregiving demands is nothing new [25]; the COVID-19 pandemic only contributed to a digital leap. The COVID-19 pandemic also resulted in a relatively low number of focus group interview participants. Only participants who had tried volunteering participated in the focus group interviews, contributing to a lack of qualitative data collected from participants who chose to not try volunteering. A future study could collect more data from participants who enroll in the program but withdraw or do not try volunteering.

Strengths of the study include the use of mixed methods and the adherence to validated recommendations for intervention development and implementation description. Another strength of the study is that three of the researchers were involved in the development and implementation of the intervention, thus possessing extensive knowledge on the intervention assessed and the focus group interview participants. However, this could have influenced the results in the focus group interviews, as the participants may have hesitated to share information about negative experiences of the intervention. On the other hand, the participants were informed about their important role as sources of information in this feasibility study to improve future interventions. Furthermore, the results from the focus group interviews are aligned with written feedback collected anonymously during the intervention (not presented here). 

## 5. Conclusions

The findings of this study imply that the Expert Caregiver intervention was feasible, as it resulted in a co-designed training course consisting of 30 h with 25 participants enrolling and 19 of those trying volunteering activities. The participants reported high scores on well-being throughout the intervention. The analysis of the focus group interviews revealed that the Expert Caregivers experienced the intervention as meaningful and offered them a sense of belonging with the other participants. Apart from using their caregiving past as an asset, the participants also took advantage of other personal skills and resources. Risks of adverse effects were related to high expectations on one’s contribution, demanding peer support recipients, poorly functioning groups, and insufficient distance to one’s caregiving past. Therefore, continuing support from intervention providers may be deemed necessary. Future studies with larger samples should investigate whether the effects of the intervention differ between subgroups of participants as well as explore the perspectives of the peer support recipients.

## Figures and Tables

**Table 1:** The intervention content and timeline.

Session	Components
Training course session 1/3 August 2019 Theme: Me as an informal caregiver then and now (4 h)	Presentation of participants and intervention From caregiver to Expert Caregiver Written promise of silence
	Optional self-studies at home in terms of literature about how to cope as an informal caregiver written by a former caregiver (4 h)
Training course session 2/3 October 2019 Theme: The everyday puzzle of an informal caregiver (camp, 5 h + 8 h)	Service, support, legislation, and rights for informal caregivers in Finland The role of the Expert Caregiver: to listen The role of the Expert Caregiver in peer group discussions Visit from a person trained in performing spokesman assignments out of personal experience
October 2019–September 2020 11 months long period of optional volunteering opportunities coordinated by the intervention providers	Agreement made between Expert Caregiver, intervention facilitator, and peer support recipient or organizer outside of the intervention to determine the timeline, content, and extent of the activity Intervention facilitator attending first meeting between Expert Caregiver and peer support recipient(s) Regular contact (about every 3 months) between intervention facilitator and Expert Caregiver (both those involved and those not involved in volunteering activities) to reflect on experiences of volunteering and to plan further activities according to the Expert Caregiver’s wishes
Training course session 3/3 December 2019 Theme: Me as an Expert Caregiver—the journey so far and what next? (5 h)	Discussions about what happens after the course Self-reflection led by a trained supervisor
Follow-up session September 2020 Theme: Coping as an Expert Caregiver (3 h)	How to take care of one’s well-being as an Expert Caregiver Self-reflection

**Table 2:** Characteristics of final 23 participants at baseline.

Variable	*n*	%	Mean	Range	SD
Age			72.52	57–82	6.93
Gender					
Female	18	78.3			
Male	5	21.7			
Education					
Lower secondary	7	30.4			
Upper secondary	16	69.6			
Household					
Alone	19	82.6			
With someone	4	17.3			
Relationship to main CR					
Spouse	18	78.3			
Parent	5	21.7			
Years since main caregiving ended	21		2.48	0–16	4.06
Years of caregiving to main CR			8.13	0–37	7.43
Age of former CR at death	21		78.29	62–101	10.15
Diagnosis of main CR					
Cognitive disorder	9	39.1			
Other	14	60.9			

CR = care recipient, SD = standard deviation.

**Table 3:** Summary of results from the analysis of the focus group interviews.

Research Focus	Main Theme	Subthemes
3.3. Practicality: Ability of participants to carry out activities	3.3.1. Individual barriers and facilitators to becoming an Expert Caregiver	*Enough distance to one’s own caregiving experience* *Current life circumstances*
	3.3.2. Competency as an Expert Caregiver	*Using new and old skills* *Relationship between Expert Caregiver and peer support recipient* *The role of the intervention organizers for feeling safe*
	3.3.3. Volunteering during COVID-19	
3.4. Practicality: Perceived effects on well-being	3.4.1. Perceived effects on the participants’ well-being and related Mediators	*Expectations influencing the experience* *The needs of caregivers as a stressor* *Volunteering as a therapeutic, meaningful or joyful activity* *A sense of belonging with the other participants*

**Table 4:** Average scores in SOC and SLWS among the participants at baseline (T1), 4 months after baseline (T2), and 13 months after baseline (T3).

Variable	T1 (*n* = 25)	T2 (*n* = 24)	T3 (*n* = 23)
SOC	67.60, 8.20, 51–82, 69	68.96, 7.79, 48–86, 69.5	68.52, 8.63, 50–81, 70
Comprehensability	24.64, 4.20, 14–32, 25	25.67, 3.63, 19–33, 26	25.43, 3.57, 17–31, 26
Manageability	20.24, 2.85, 14–25, 21	20.75, 2.97, 13–26, 21	20.52, 3.93, 9–25, 22
Meaningfulness	22.72, 3.08, 18–28, 23	22.54, 2.99, 16–27, 22.5	22.57, 3.30, 14–26, 23
SLWS	25.92, 5.24, 12–33, 26	26.38, 5.77, 15–34, 29	26.61, 4.92, 16–34, 28

The table shows mean, standard deviation, range, and median.
